# Contribution of Adipose Tissue Oxidative Stress to Obesity-Associated Diabetes Risk and Ethnic Differences: Focus on Women of African Ancestry

**DOI:** 10.3390/antiox10040622

**Published:** 2021-04-19

**Authors:** Pamela A. Nono Nankam, Télesphore B. Nguelefack, Julia H. Goedecke, Matthias Blüher

**Affiliations:** 1 Helmholtz Institute for Metabolic, Obesity and Vascular Research (HI-MAG) of the Helmholtz Zentrum München at the University of Leipzig and University Hospital Leipzig, 04103 Leipzig, Germany; Matthias.Blueher@medizin.uni-leipzig.de; 2 Laboratory of Animal Physiology and Phytopharmacology, Faculty of Sciences, University of Dschang, Dschang 96, Cameroon; Telesphore.Nguelefack@univ-dschang.org; 3 Non-Communicable Diseases Research Unit, South African Medical Research Council, Cape Town 19070, South Africa; Julia.Goedecke@mrc.ac.za; 4 Medical Department III—Endocrinology, Nephrology, Rheumatology, University of Leipzig Medical Center, 04103 Leipzig, Germany

**Keywords:** obesity, adipose tissue, oxidative stress, ethnicity, metabolic risks

## Abstract

Adipose tissue (AT) storage capacity is central in the maintenance of whole-body homeostasis, especially in obesity states. However, sustained nutrients overflow may dysregulate this function resulting in adipocytes hypertrophy, AT hypoxia, inflammation and oxidative stress. Systemic inflammation may also contribute to the disruption of AT redox equilibrium. AT and systemic oxidative stress have been involved in the development of obesity-associated insulin resistance (IR) and type 2 diabetes (T2D) through several mechanisms. Interestingly, fat accumulation, body fat distribution and the degree of how adiposity translates into cardio-metabolic diseases differ between ethnicities. Populations of African ancestry have a higher prevalence of obesity and higher T2D risk than populations of European ancestry, mainly driven by higher rates among African women. Considering the reported ethnic-specific differences in AT distribution and function and higher levels of systemic oxidative stress markers, oxidative stress is a potential contributor to the higher susceptibility for metabolic diseases in African women. This review summarizes existing evidence supporting this hypothesis while acknowledging a lack of data on AT oxidative stress in relation to IR in Africans, and the potential influence of other ethnicity-related modulators (e.g., genetic-environment interplay, socioeconomic factors) for consideration in future studies with different ethnicities.

## 1. Introduction

One of the primary functions of adipose tissue (AT) is the storage of triglycerides during positive calorie balance and the release of free fatty acids (FFAs) in periods of energy demand. Subcutaneous adipose tissue (SAT) is the largest adipose depot, representing 80–90% of total fat mass [1] and the most important quantitative contributor to lipid storage/release and endocrine function [2]. In humans, the abdominal, subscapular and gluteo-femoral regions typically represent the largest fat depots [1,3,4]. Among abdominal AT, visceral adipose tissue (VAT) is distributed in the abdominal cavity around intraabdominal organs and represents 10–20% of total body fat in men and 5–10% in women [1]. White AT can also be found in organs including the liver, skeletal muscle (intra- and extra-myocellular fat), heart (epicardial fat), and pancreas where it may serve specialized functions related to these tissues and is usually referred to as ectopic fat [3,4].

Although representing only one-third of the cell types in AT, adipocytes are the principal protagonists in the process of lipid storage/release [5]. Excess lipid storage in adipocytes during positive energy balance results in an increase in cell number (hyperplasia) and/or cell size (hypertrophic obesity), and consequently, an increased AT volume [6,7]. As a major supplier of FFAs to other organs in the post-absorptive state, enlarged hypertrophic AT may result in increased FFAs released into the circulation due to increased basal lipolysis [2]. In addition to metabolite flux, adipokine secretion is altered in hypertrophic obesity [7,8]. Adipokines are AT-derived bioactive molecules involved in the regulation of several cellular and metabolic mechanisms [9]. Dysregulation in metabolite release and adipokine secretion has been shown in the development of obesity-associated metabolic dysfunction such as insulin resistance (IR) and type 2 diabetes (T2D), which highlights the influence of AT function on whole-body metabolism [2,10].

**Figure 1:**
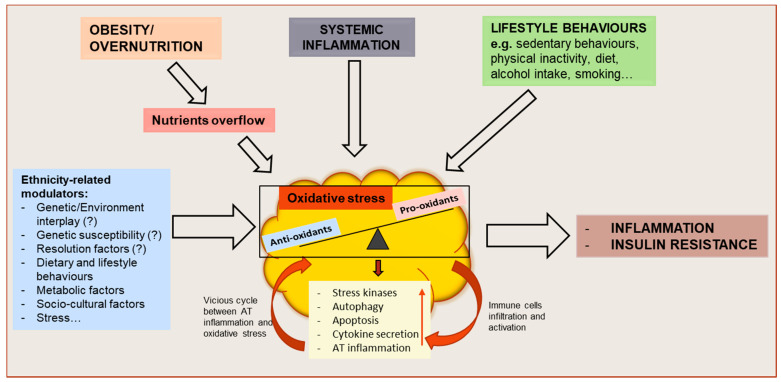
Schematic representation of oxidative stress drivers and metabolic consequences on adipose tissue (AT) function and whole-body metabolism. Obesity or overnutrition may result in nutrients overflow to AT, resulting in adipocyte hypertrophy and AT hypoxia which might induce an oxidative stress state in the tissue. Systemic inflammation, as well as behavioral factors, may also contribute to the disruption of the redox equilibrium of AT. As a result, the activation of stress signaling pathways contribute to increasing autophagy and apoptosis, dysregulated adipokine secretion and AT inflammation. The resulting functional alterations may further impair AT function by causing an increased attraction, infiltration and activation of immune cells and increased AT inflammation, creating a vicious cycle between AT oxidative stress and inflammation, and leading to whole-body metabolic dysfunction. These mechanisms might be influenced by ethnicity-related modulators.

Impaired AT expandability, potentially leading to AT dysfunction in obesity is an accepted theory for the development of obesity-associated metabolic disorders in some, but not all individuals [10]. AT dysfunction may be initiated by pathological mechanisms such as hypoxia that propagate several types of AT stresses including inflammatory, metabolic, endoplasmic reticulum and oxidative stress [10,11]. Oxidative stress has been demonstrated in individuals with obesity and is reflected by elevated markers of reactive oxygen species (ROS) such as isoprostanes, circulating thiobarbituric reactive acid substances (TBARS) or malondialdehyde (MDA), lipid hydroperoxides (LOOH), as well as reduced antioxidant defense system [12,13,14]. Oxidative stress has also been associated with systemic inflammation [12,15], impairment of glucose uptake into adipocytes [16], and decreased insulin secretion from pancreatic β-cells [17], supporting its critical role in the pathogenesis of T2D. Besides, increased oxidative stress in accumulating AT has been associated with dysregulation of adipokine secretion and IR in individuals with obesity, while both increased systemic and AT oxidative stress have been associated with obesity-induced inflammation [12,18]. Therefore, understanding the relationship between obesity-induced oxidative stress and the associated metabolic disorders is of relevance for the elucidation of disease mechanisms and identification of treatment targets (Figure 1).

The incidence of obesity and associated co-morbidities, as well as the degree of translation of adiposity into cardio-metabolic diseases, vary among different ethnicities [19,20,21,22,23,24,25]. In this regard, a higher prevalence of obesity is reported in African populations as compared to populations of European ancestry, mainly driven by higher rates among African women [26]. Moreover, it has been shown that populations of African ancestry present with hyperinsulinemia, are more insulin resistant and at greater risk for T2D than populations of European ancestry [24,26,27]. This is concomitant with differences in AT morphology and function between these two ethnic groups suggesting that ethnic-specific AT function may be partly involved in the development of obesity-associated metabolic dysfunction. Indeed, we previously showed that some pathways related to AT dysfunction might be ethnically distinct [28,29], with higher markers of AT hypoxia (e.g., hypoxia-inducible factor 1 -HIF-1α) in individuals of African compared with European ancestry [29]. Higher exposure to hypoxia may increase the levels of oxidative stress [10,30] which have been associated with increased circulating FFAs concentrations and reduced insulin sensitivity in black Africans, but not white Europeans [31]. Therefore, ethnic differences in insulin sensitivity and T2D risk could be associated with differentially increased oxidative stress [26]. Compared to individuals of European ancestry, individuals of African ancestry exhibit higher levels of systemic oxidative stress markers [31,32,33,34]. However, there is a dearth of studies that have explored differences in AT oxidative stress between different ethnicities. Notably, the research term “oxidative stress and adipose tissue” in the PubMed NIH database gave over 2600 outputs while the research term “oxidative stress, adipose tissue and ethnicity” only gave 11 results (searched on 1 February 2021). This underlines the lack of evidence regarding the ethnic-specific difference in AT oxidative stress markers. Furthermore, whether an increased oxidative stress state in AT might be partly involved in the higher metabolic risk observed in Africans with obesity is not known. This review will focus on oxidative stress as one of the mechanisms potentially involved in ethnic-specific differences in obesity-associated T2D risk. However, it should be noted that these ethnic-specific discrepancies might not be limited to physiological factors. Rather, other factors such as lifestyle behaviors, environmental and stress factors, socioeconomic and cultural backgrounds, as well as different access to healthcare might significantly influence the reported/observed ethnic-related susceptibility and physiological differences [35] (Figure 1).

## 2. Definition of Oxidative Stress

**Figure 2:**
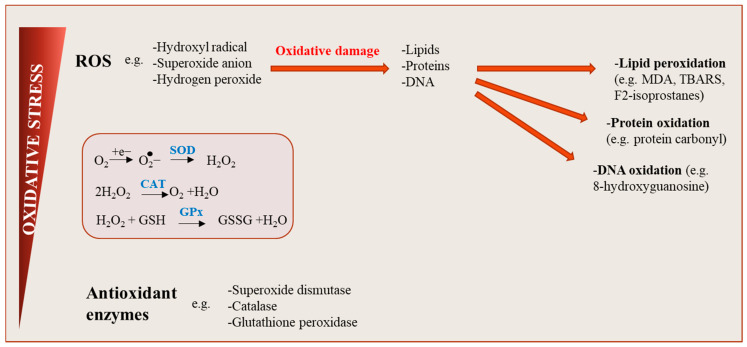
Oxidative stress: an imbalance between reactive oxygen species (ROS) production and antioxidant defenses. ROS are generated during cellular metabolism when the chemical reduction of oxygen forms unstable free radicals. Several molecule types including lipids, proteins or nucleic acids can be oxidized or nitrated, and the resultant product, when accumulated in cells over time become harmful, affecting cell signaling pathways and tissue function. Physiological levels of ROS are conserved by the action of antioxidants, maintaining a redox balance in cells.

Oxidative stress generally refers to an imbalance between the production of pro-oxidant substances (i.e., free radicals, ROS and/or reactive nitrogen species (RNS)) and the antioxidant defense system [12]. ROS are generated during cellular metabolism when the chemical reduction of oxygen forms unstable free radicals, characterized by an unpaired electron (e.g., superoxide (O_2_^−^), hydrogen peroxide (H_2_O_2_), hydroxyl radical (OH^−^); Figure 2) [36]. Proteins, DNA, lipids and sugars are cellular targets for chemical modification via oxidation, nitration or nitrosylation, resulting in cell damage and/or changes in cell signaling pathways, thereby, affecting tissue function [37,38,39]. Under normal conditions, these oxidizing agents are essential for physiological functions such as regulation of gene expression, cellular growth, cell signaling and infection defense [12,36,40]. Indeed there is increasing evidence that ROS do not only cause oxidative stress, but may function as signaling molecules that promote health by preventing or delaying several chronic diseases, and ultimately extend lifespan [41]. This concept is also known as “mitohormesis” [42,43]. Eventually, increased oxidative stress in AT may contribute to increasing autophagy and apoptosis [44,45,46]. To control such dysregulation in cells, physiological levels of ROS are conserved by the action of antioxidants from enzymatic or non-enzymatic sources [12]. Antioxidants are important to prevent oxidizing damage of ROS and maintain a redox balance in cells. This action might be exerted through their capacity to inhibit ROS formation and initiate bio-oxidative processes, to scavenge ROS and free-radicals, block oxidation propagation and repair oxidized molecules or ROS-damaged cells [12,47]. The main cellular antioxidant enzymes involved in these processes are superoxide dismutase (SOD), catalase and glutathione peroxidase (GPx) [47,48]. These enzymes are responsible for the conversion of superoxide radicals to hydrogen peroxide (mainly SOD) and subsequently to water and oxygen (mainly catalase and GPx) [48]. SOD represents the primary antioxidant protection against the harmful effects of ROS and is mostly found in cell cytosol (SOD1) and mitochondria (SOD2), while catalase can be found in all tissues utilizing oxygen, mainly abundant in peroxisomes and mitochondria [48]. GPx acts principally by inhibiting lipid peroxidation, and by neutralizing hydrogen peroxide in the mitochondria using the substrate glutathione [46,47]. However, excessive production of reactive species can result in depletion of the antioxidant defense system and increase cell vulnerability to oxidative damage (Figure 2) [40]. Consequently, long-term exposure to elevated ROS may contribute to the progression of chronic inflammation and IR by the reduction of insulin signaling and impairment of glucose and lipid metabolism in tissues [16,18].

Oxidative stress can be evaluated in various tissues and body fluids using several techniques. However, due to a short half-life, reactive species are unstable molecules to measure. Accordingly, the quantification of oxidative stress in humans is mainly via indirect evaluation [49]. Hence, rather than the direct measure of ROS, the products from the oxidative (or nitrated) damage are assessed (e.g., malondialdehyde, F2-isoprostanes), as these products are more stable than reactive species. Alternatively, oxidative stress can be indirectly evaluated by measuring ROS-producing enzymes or antioxidant concentrations, expression or activities [49].

## 3. Oxidative Stress in Obesity

Given the major health burden caused by the double pandemic of obesity and T2D, it is important to understand the causal mechanisms underlying their relationship, as well as the implication of oxidative stress in this association. Oxidative stress has been evidenced during the development of obesity with elevated levels (urinary, systemic and/or tissue-specific) of biomarkers such as 8-epi-Prostaglandin F2α (8-iso-PGF2α), 4-Hydroxynonenal (4-HNE) and MDA in children and adults [50,51], insulin-sensitive and insulin-resistant patients with obesity [52,53,54,55,56,57]. Moreover, dysregulation in antioxidant defense has been shown, as well as inverse associations between antioxidant capacity and body fat percentage. This suggests that the degree of adiposity affects antioxidant enzyme activities [57,58], and/or vice versa [59,60]. Accordingly, Elrayess et al. showed increased antioxidant enzymes (SOD, catalase, thioredoxin, peroxiredoxin) in preadipocytes treated with 4-HNE [52], while Jankovic et al. found lower levels of glutathione in VAT and SAT of women with obesity compared to normal-weight women [61]. The increase of antioxidant enzymes in response to high ROS concentrations might be a compensatory response during early-stages of obesity development, in order to maintain the oxidative balance until the antioxidant capacity is depleted [57]. A sustained increase in the endogenous activity of antioxidant enzymes can reduce the incidence of oxidative stress and associated metabolic disorders by regulating ROS production [62,63]. For instance, antioxidant treatment improves insulin function in people living with diabetes [64]. In contrast, individuals with a genetic predisposition for low catalase activity (e.g., acatalasemia, catalase mutations) are at higher risk of T2D [65]. Moreover, the deletion of endogenous catalase (*Cat^−/−^* mice) results in the development of liver steatosis and inflammation both on chow and high-fat diets [59,66]. Furthermore, *Cat^−/−^* mice show a pre-diabetic phenotype characterized by impaired glucose tolerance and increased fasting serum insulin [59]. These and several other studies provide evidence for tight relationships between oxidative stress, central obesity and excess fat accumulation, as well as associated metabolic disorders such as inflammation, IR and T2D [18,50,67,68,69,70,71,72].

The occurrence of oxidative stress in obesity is further demonstrated by an upregulated expression of the ROS-producing enzyme NADPH (nicotinamide adenine dinucleotide phosphate) oxidase (NOX) in AT of patients with obesity and IR [18,73,74,75]. Notably, oxidative stress in AT may be distinct for specific fat depots. For instance, higher concentrations of hydrogen peroxide, catalase and SOD activities were shown in VAT (and not in SAT) from men with central obesity, compared to normal-weight men [76]. It was also shown that NOX is expressed to a higher extent in human VAT compared to SAT [61]. Hence, oxidative stress could be involved in the detrimental effects of fat accumulation in VAT compared to SAT, as reported in several studies [10,77,78,79,80,81]. Interestingly, NOX inhibition improves adipokine secretion and increases insulin sensitivity via restoration of normal ROS production in adipocytes from individuals with obesity [18]. Therefore, oxidative stress develops in adipocytes with the development of obesity and may contribute to further impairment of AT function.

The activation of NOX is one of the principal pathways of ROS generation in AT [18,68]. NOX generates reactive species by transferring electrons from intracellular NADPH to the membrane and coupling these to molecular oxygen to produce superoxide anions, which might be further transformed into hydrogen peroxide [82,83,84]. Increased *NOX4* expression in AT of obese mice, parallel with altered NADPH pathways and a reduced expression of antioxidant enzymes (SOD, catalase and GPx), results in higher lipid peroxidation and production of hydrogen peroxide [18]. Moreover, in a high-fat diet, increased ROS production in adipocytes is mediated through higher NOX4 expression and activity [74,85]. These findings support the role of NOX signaling pathways in AT oxidative stress and ROS generation, especially under conditions of excessive nutrient availability and fat accumulation [18,85].

One of the cellular targets for ROS production by NOX signaling pathway is the mitochondrion [84]. This organelle is mainly responsible for cellular energy production by oxidative phosphorylation. During oxidative phosphorylation, a small excess of electrons causes a reduction of an oxygen molecule, generating a potentially toxic free radical such as superoxide [84,86]. Given the excess nutrient availability to adipocytes in obese AT, the mitochondrial activity significantly increases, resulting in increased ROS production [87]. For instance, FFAs can promote the generation of oxygen in the mitochondrial electron transport chain by stimulating the production of reactive intermediates through protein kinase C-dependent activation of NOX [88]. Moreover, glucose overload in cells can lead to the overproduction of NADH, resulting in increased electron leakage from the mitochondrial membrane and production of superoxide [89]. Besides the excessive availability of energy substrates, higher oxidative stress in AT has been shown to induce mitochondrial dysfunction [90,91]. This exacerbates oxidative stress in AT by altering the regulation of free radical production in mitochondria and has been linked to defective fatty acid oxidation, dysregulation of adipokine secretion, and alteration of glucose homeostasis [89].

## 4. Obesity-Induced AT Dysfunction and Oxidative Stress

**Figure 3:**
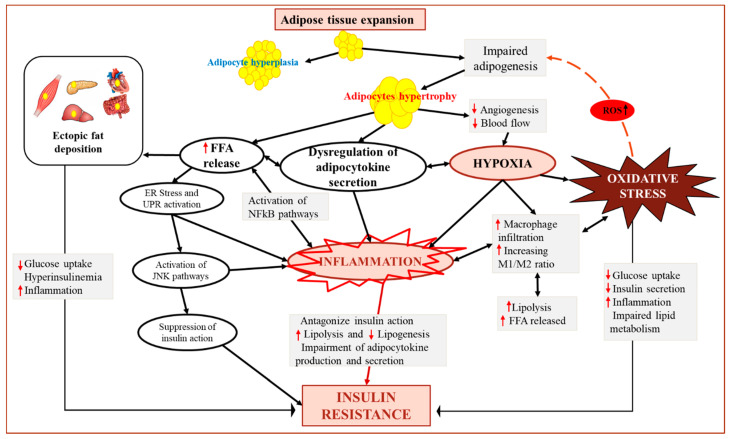
Adipose tissue expandability: proposed mechanisms whereby impaired adipogenesis during AT expansion may link oxidative stress to insulin resistance (IR). The most accepted mechanisms implicated in the impairment of AT function during excess fat accumulation include impaired adipogenesis and adipocyte hypertrophy, followed by increased FFAs release and ectopic fat deposition, dysregulation of adipokine secretion, increased hypoxia and AT cellular stresses such as oxidative stress. These mechanisms contribute in concert to the establishment of a pro-inflammatory state in AT, interfering with the insulin signaling pathway and leading to peripheral and systemic insulin resistance. Via elevated ROS production, oxidative stress may further impair AT function initiating a vicious cycle between AT expansion and IR. Abbreviations: ER: endoplasmic reticulum; FFA: free fatty acids; UPR: unfolded protein response; JNK: Jun N-terminal kinase; NFkB: nuclear factor-kappa B.

Oxidative stress is one of the characteristics of impaired AT function during excessive body fat accumulation and is suggested to play a role in the development of obesity-associated IR [70,92,93]. An impaired ability of precursor stem cells to enter adipogenesis (adipocyte development process) may result in adipocyte hypertrophy and impaired extracellular matrix remodeling [18,94,95,96]. This might be followed by a reduction in oxygen supply to adipocytes, setting up a local hypoxic environment and contributing to the establishment of pro-inflammatory and oxidative stress states in AT [97,98]. Chronic hypoxia may affect intracellular insulin signaling pathways and subsequently leads to peripheral and systemic IR (Figure 3) [18,94,96,97]. Indeed, hypoxia in hypertrophic adipocytes has been shown to stimulate pro-inflammatory adipokine secretion (such as angiopoietin-like protein 4, IL-6, leptin and macrophage migration inhibitory factor (MIF-1)) and induce oxidative and endoplasmic reticulum (ER) stresses (Figure 3) [99]. Notably, hypoxia increases macrophage infiltration, fibrosis and oxidative stress [96,100], causing cell damage and activation of stress signaling pathways [101]. Concurrently, recruited and activated immune cells (e.g., T cells and macrophages) may also generate NOX2-derived ROS in the intermediate stage of obesity [102]. In addition to hypoxia and immune cells infiltration, lipid accumulation in adipocytes leads to the generation of excessive ROS, which in turn impairs the healthy expansion of AT [103], resulting in inflammatory reactions and IR [18,99]. Furthermore, elevated FFAs release, resulting from increased lipolysis can sustain oxidative stress by activating NOX followed by excess ROS production [18]. Therefore, oxidative stress could be both a consequence of obesity-associated AT dysfunction and a factor sustaining the impairment of AT function (Figure 3).

Studies that have explored the influence of oxidative stress on adipogenesis presented conflicting data. For instance, AT hypoxia and oxidative stress have been shown to inhibit adipocyte differentiation and proliferation through the downregulation of peroxisome proliferator-activated receptor-gamma (*PPARγ*) and adiponectin by hypoxia-inducible transcription factor, HIF-1 [18,99]. Higher levels of 4-HNE (bio-reactive aldehyde) inhibits human SAT preadipocyte growth and de novo lipogenesis by upregulating the anti-adipogenic gene *FABP4* and downregulating the adipogenic genes *FASN* and *SREBF1* [52,103]. This contributes to the impairment of insulin signaling by dephosphorylating the insulin receptor substrate-1 (IRS1) [52]. Moreover, prolonged exposure of adipocytes to ROS impairs insulin-induced activation of PI3-kinase and Akt, insulin-stimulated lipogenesis, glucose transporter (GLUT)-4 translocation to the plasma membrane and glucose uptake [16,104]. In contrast, dysregulated adipogenesis by ROS was demonstrated by increased proliferation of adipose-derived stem cells (ASCs) and preadipocyte differentiation [105,106], followed by a reduction of NOX4 content [107,108]. Moreover, hypoxia induces increased ASCs expression of vascular endothelial growth factor (VEGF), which in turn stimulates adipocyte proliferation [109]. Noteworthy, non-coordinated adipogenesis and angiogenesis may result in the impairment of recruitment and differentiation of ASCs, contributing to adipocyte hypertrophy [110]. These contradictory data might result from differences in study designs (in vitro vs. in vivo), studied species (animal vs. humans), experimental techniques and ROS conditions used in the cell culture experiments. Another reason could be the difference in ROS concentration as per the mitohormesis theory, stating that different amounts of ROS may explain their dual role in health and disease [41]. In fact, “lower” concentrations of ROS may promote health due to their essential role as signaling molecules, while “higher” concentrations, over a prolonged period may become deleterious, causing cellular and systemic damage [42].

## 5. Dysregulation of Adipokine Secretion in Response to AT Oxidative Stress

Dysregulated adipokine secretion and inflammation might be a cause and consequence of oxidative stress in AT [111]. Adipokines are involved in the regulation of adipogenesis, fat distribution, immune cell infiltration, adipocyte metabolism, as well as inflammation, and may link obesity-related oxidative stress to IR [9]. Leptin, one of the first discovered adipokines, is almost exclusively secreted by adipocytes, significantly upregulated with obesity and fat accumulation [112,113] and related to the development of the metabolic syndrome [9,114]. In addition to its role as a satiety signal and potent mediator of IR, leptin can induce oxidative stress by stimulating fatty acid oxidation in mitochondria [115], activate NOX and induce the production of hydrogen peroxide and hydroxyl radicals [116], stimulate the proliferation of monocytes and macrophages in AT [114,117]. In this context, higher leptin levels have been associated with increased production of TNF-α and IL-6, increased activity of NOX and the production of ROS [118,119].

AT inflammation and oxidative stress are tightly interrelated mechanisms with crosslinks between their respective signaling pathways, contributing to obesity-associated IR [74,120] and AT dysfunction [121]. During AT accumulation, macrophage infiltration into AT plays a vital role in regulating inflammation by their ability to shift adipocyte secretory profile towards a pro-inflammatory condition (T-helper 1 subtype) [122]. However, macrophages can also produce ROS (e.g., O_2_^−^, H_2_O_2_ and OH^−^), which provides positive feedback to upregulate T-helper 1 cell activation and further sustain a pro-inflammatory state [123]. Moreover, increased NOX2 activity following macrophage-induced ROS production may subsequently dysregulate the expression of inflammatory adipokines (e.g., reduced adiponectin, increased plasminogen activator inhibitor-1 (PAI-1), monocyte chemoattractant protein 1 (MCP-1) and IL-6) and decrease the production of antioxidant enzymes [18,64,74,124]. ROS and by-products of lipid peroxidation such as MDA further induce the attraction, infiltration and activation of macrophages into AT and inflammation [18,74].

In addition, oxidative stress may directly impair insulin signaling pathways. The accumulation of ROS and modified ROS-damaged proteins can activate stress signaling pathways such as the serine/threonine kinase, c-Jun-N-terminal kinase (JNK) causing the IRS-phosphorylation on inhibitory serine residues [125,126,127]. Moreover, hydrogen peroxide can activate the phosphorylation of IĸB via p38 mitogen-activated protein kinase (MAPK) inhibition, followed by the activation of NFĸB (nuclear factor kappa-light-chain-enhancer of activated B cells) [125,126]. Through these effects on the intracellular insulin signaling cascade, increased ROS may impair lipid storage, induce inflammation, impair insulin sensitivity, and thereby exacerbate the oxidative stress state via a positive feedback loop. The resulting vicious cycle potentially instigates whole-body IR by affecting peripheral organs’ function, supporting the implication of AT oxidative stress in the development of obesity-associated metabolic dysfunction.

## 6. Role of Fat Distribution in Obesity-Associated Oxidative Stress

Body fat distribution is a stronger determinant for the risk to develop obesity-associated complications than total adiposity. While central fat accumulation (both VAT and abdominal SAT) is associated with increased risk of metabolic diseases, peripheral fat distributed in the lower body is believed to have a protective role against these disorders [30,77,128,129,130,131]. Interestingly, investigating the adipose-depot specific associations with systemic oxidative stress, Kelli et al., [68] proposed that VAT accumulation is an independent contributor to detrimental cardiometabolic profile through the modulation of systemic oxidative stress. Indeed, changes (increase) in android fat, but not gynoid fat distribution or BMI over a year correlated with reduced antioxidant capacity (assessed by plasma glutathione level) after adjustment for cardio-metabolic risk factors [68]. Similarly, VAT was more highly associated with urinary isoprostanes than abdominal SAT in adults with obesity [132]. Remarkably, a study in Asians with obesity showed that compared to abdominal SAT-derived adipose precursor cells, VAT-derived cells had higher ROS concentrations and lower antioxidant gene expression [133]. Furthermore, higher expression of endothelial NOS (responsible of nitric oxide production), was found in VAT compared to abdominal SAT in European men [134] and European and African women with obesity [135]. These findings suggest that excessive VAT accumulation is associated with an unfavorable oxidative stress profile, supporting their role in the deterioration of metabolic health in individuals with central obesity.

### Role of Ethnicity in Oxidative Stress Regulation

Body fat distribution patterns vary between ethnicities and might partly explain ethnic differences in obesity-associated cardio-metabolic risks. Indeed, it has been extensively shown that in Africans, AT is mainly distributed in peripheral SAT, with relatively low VAT (for the same BMI) than other ethnic groups including populations of European descent and Hispanic Americans [19,20,21,22,23,25,27,29,128,131,136,137,138,139]. However, despite greater peripheral SAT and less VAT (seemingly “favorable” body fat distribution pattern), individuals of African ancestry unexpectedly present with lower insulin sensitivity and are at higher risk for metabolic diseases than those of European ancestry [19,21,23,24,26,128,130,139]. The phenotype of low insulin sensitivity despite lower VAT and greater gluteal SAT in African women may be associated with changes in SAT function during progressive fat accumulation [28,30,128,140,141]. However, the specific pathways driving the associations in African women are not fully understood. For instance, we previously showed higher inflammation in the gluteal SAT compared to abdominal SAT of black African women, but this was not significantly associated with their reduced insulin sensitivity [28]. Only a few studies have examined the relationship between SAT function and IR in this population, which warrants further investigations.

More studies have examined ethnic differences in systemic oxidative stress and shown this to be higher in Africans compared to other ethnic groups matched for BMI [31,32,33]. Strikingly, African ethnicity is a proposed independent risk factor for enhanced oxidative stress and inflammation [33]. Indeed, increased circulating protein carbonyls were reported in black African, but not in white European women (non-diabetic; overweight and obese) [31]. This was concomitant with higher concentrations of circulating FFAs, which correlated with reduced insulin sensitivity independently of body fat percentage in African women [31]. In addition, higher circulating lipid hydroperoxide was shown in BMI-matched diabetic patients of African-Caribbean compared to European descent [32]. Furthermore, lower circulating levels of the antioxidant glutathione were found in African compared to European descent men and women with or without metabolic syndrome [34]. Of note, these studies mainly evaluated systemic oxidative stress, and there is a lack of studies reporting ethnic differences in the associations between AT oxidative stress and cardio-metabolic risk factors.

## 7. Adipose Tissue Oxidative Stress as a Risk Factor of Metabolic Dysfunction in Africans

Considering systemic oxidative stress as a component of cardio-metabolic risk, it can be postulated that the ethnic-specificity in the occurrence of oxidative stress may support, at least in part, the ethnic disparities in obesity-associated comorbidities and the higher susceptibility to metabolic disorders in Africans. However, there is a lack of evidence for the proposed relationship between oxidative stress and obesity-associated metabolic risk (or dysfunction) in Africans. In particular, there is no direct experimental evidence for higher AT oxidative stress in relation to adiposity and the metabolic profile in Africans. The majority of studies evaluating AT oxidative stress markers have been conducted in European or non-African populations [52,61,91,142]. To the best of our knowledge, only one study in Egyptians with central obesity reported associations between AT oxidative stress and measures of adiposity and IR [76]. This study showed higher levels of hydrogen peroxide in VAT of men with obesity compared to normal-weight men, which were positively associated with IR [76]. Further, higher catalase activity in VAT was an independent determinant of IR in this study [76]. Notably, we recently showed an improvement of systemic oxidative stress in response to exercise training in black African women with obesity, but this was not correlated with the reported improvement in insulin sensitivity [143]. Interestingly, we did not find significant associations between SAT oxidative stress markers (eNOS, SOD and catalase genes) and whole-body insulin sensitivity. We rather showed positive correlations between increased inflammatory markers and increased SOD and catalase expression in gluteal SAT [143]. These data suggest that associations between SAT oxidative stress and IR in black African women might be mediated via the complex interrelation with inflammation, mitochondrial dysfunction or hypoxia, which were not directly assessed in our study [143]. Taken together, more studies on AT-related disease mechanisms in individuals of African ancestry and involving larger sample sizes are needed. Moreover, future studies should be designed to decipher whether and how AT oxidative stress may underly the highly variable associations between obesity and its related diseases in Africans.

## 8. Mechanisms Contributing to Higher Measures of Oxidative Stress Markers in Africans

Additional factors contributing to the generation of ROS in obesity might be considered to explain the high rates of oxidative stress alongside the high risk for metabolic diseases in Africans. These factors include, among others, hyperglycemia, impaired AT and mitochondrial function, elevated FFAs levels, inflammation, hyperleptinemia, or even dietary intake and environmental factors [35,57,89].

### 8.1. Hyperglycemia

Hyperglycemia is a major risk factor for T2D, being itself one of the major complications of obesity. Individuals of African ancestry (e.g., black South Africans and African Americans) have higher diabetes prevalence [144,145] and are consequently more hyperglycemic compared to their counterparts of European ancestry, independently of body fat percentage or BMI [24,26,138]. Hyperglycemia can induce and/or increase oxidative stress [89,146] by increasing ROS production through the proton electromechanical gradient generated by the mitochondrial electron transport chain [147] or the transition metal-catalyzed autoxidation of free glucose, or by compromising antioxidant defenses [148]. This promotes the formation of advanced glycosylation end products (AGEs) and protein kinase-C activation [147]. AGEs can further react with AGE receptors, present on the surface of monocyte-derived macrophages, endothelial cells, and smooth muscle cells [148]. This induces oxidative stress and the activation of the transcription factor NFkB which initiate downstream inhibitory signaling pathways [148].

### 8.2. Adipose Tissue Function

In addition to hyperglycemia, AT dysfunction might cause higher measures of oxidative stress in Africans. Obesity impairs SAT adipogenesis and storage capacity to a greater extent in women of African ancestry compared to European ancestry, which correlates with reduced insulin sensitivity and increased risk for T2D [128]. In addition, gluteal AT gene expression signatures differed with ethnicity and may contribute to the heterogeneous ethnicity-related correlation between obesity and associated IR and cardiometabolic risk [128]. Specifically, reduced expression of PPARɤ, SREBP1, FASN, FABP and adiponectinhave been shown in gluteal [128] and abdominal SAT (PPARɤ, adiponectin, lipin-1β, SCD-1, CD36) [149] in African compared to European ancestry women with obesity. This suggests an ethnic-specific difference in the association between obesity, adipogenesis and insulin sensitivity. Moreover, black African women present with higher inflammatory profiles (CCL2, CD68, TNF-α, MIF and CSF-1 genes) in both abdominal and gluteal SAT compared to their white European counterparts, independent of total adiposity and VAT [28]. This is accompanied by a lower adiponectin gene expression and higher expression of inflammatory cytokines, macrophage markers and higher leptin expression in the gluteal than abdominal SAT of black African women [28]. In addition, lower circulating adiponectin concentrations, higher leptin levels and circulating inflammatory parameters (e.g., C-reactive protein, IL-6, MCP-1) were shown in women of African ancestry compared to Hispanic or European women [137,150]. Hyperleptinemia can reduce antioxidant activity (e.g., paraoxonase-1), increase ROS production and AT inflammation. AT inflammation, in turn, is a significant source of oxidative stress, hyperglycemia and immune cell infiltration ultimately leading to ROS formation [57,89,116]. Noteworthy, based on evidence of lower levels of circulating PAI-1 (stimulator of angiogenesis) in African compared to European and Hispanic women [151], Goedecke et al., postulated that higher levels of hypoxia may increase the expression of HIF-1 in AT, resulting in higher levels of inflammation and oxidative stress in African women [29,30]. Given the association between AT dysfunction (especially impaired adipogenesis and increased adipocytes hypertrophy, hypoxia, AT inflammation) and oxidative stress, we hypothesize that ethnic differences in AT oxidative stress related to obesity contribute to the higher metabolic risk in Africans.

### 8.3. AT Storage and Elevated Lipid Levels

One of the immediate consequences of a dysregulated adipogenesis and the resulting impaired adipose storage capacity is increased FFAs released into the circulation. Elevated circulating FFAs concentrations contribute to higher ROS production and increase oxidative stress [57,89]. It has been shown that African women have higher fasting circulating free fatty acids than their European counterparts [19]. Furthermore, acute hyperlipidemia increased systemic oxidative stress (F2-isoprostanes) to a greater extent in individuals of African descent compared to those of European descent [152]. However, elevated circulating FFAs might not only be derived from a defect in AT function but may also be associated with increased dietary fat intake.

### 8.4. Environmental, Socioeconomic and Lifestyle Factors

Environmental (e.g., pollution, exposure to heavy metals, pesticides, stress, physical environment) and lifestyle (e.g., smoking, alcohol intake, physical inactivity, dietary intake) factors are determinants in the development of obesity and related comorbidities and can promote oxidative stress [153,154]. Environmental factors could also impact behavioral (e.g., unhealthy diet, insufficient or no exercise and sleep quality …) and mental health [35], further disturbing the oxidative balance [155]. For instance, lower plasma antioxidant capacity has been associated with poor sleep quality and higher depression [156]. Moreover, higher lipid peroxidation positively correlated with sedentary behavior and negatively with higher caloric expenditure [157].

Socioeconomic status is a key determinant of obesity particularly among black African women [158] and also influences disease risk by promoting oxidative stress [155]. Accordingly, urinary concentrations of 8-iso-PGF2α were shown to increase with lower socioeconomic status in women [159]. Furthermore, racial inequalities in socioeconomic status with a disadvantage among African Americans compared to European Americans have been associated with higher obesity rates and obesogenic environments [160,161]. Therefore, the physical environment and cultural or historic background may partly drive physiological mechanisms resulting in increased obesity prevalence in black African populations. These factors should be considered when investigating the mechanisms underlying the relatively higher risk of obesity-related cardiometabolic diseases among populations of African ancestry compared to populations of European ancestry.

Differences in socioeconomic status in distinct groups may also affect the quality of food consumption. The dietary intake of some macronutrients can influence systemic oxidative stress [57,162]. Accordingly, rats fed with a high-fat diet exhibited an increased myocardial lipid peroxidation and TBARS concentrations, mainly attributed to higher myocardial lipid content [163]. Moreover, a reduced antioxidant defense (erythrocyte SOD and GPX activities) was shown in rats after a high-fat, high-calorie diet [164]. In individuals with obesity, the consumption of conjugated linolenic acid (polyunsaturated fatty acid mainly derived from dairy products and meat from ruminant animals) can elevate oxidative stress by increasing urinary 8-epiPGF2α [57]. African women were shown to have higher dietary fat intake than their European counterparts [21]. Similarly, we [165] and others [166] showed high consumptions of fat and high glycemic carbohydrates in Africans. The consumption of high-fat and high-carbohydrate diets induce a significant increase in oxidative stress through the activation of NOX and NFkB pathways [162]. Therefore, dietary habits may potentially contribute to the pathogenesis of obesity-associated IR and enhanced oxidative stress in populations of African ancestry [30]. However, future investigations should explore this as it has not been directly studied.

### 8.5. Impaired Mitochondrial Function

During excess food consumption, hyperglycaemia and elevated FFAs, the mitochondrial energetic efficiency and ROS production increase [26]. We recently showed that higher gSAT mitochondrial respiratory capacity was associated with higher gluteal fat accumulation in a cohort of African women [167]. Similarly, the resulting higher production of hydrogen peroxide correlated with lower insulin sensitivity in these women [167]. Elevated mitochondrial efficiency and the resulting ROS overproduction, could be associated with metabolic differences between individuals of African and European ancestry [26]. This might be another factor contributing to the greater risk of IR and T2D in Africans.

## 9. Conclusions

This review discusses the origin, role and complexity of oxidative stress in the pathogenesis of obesity-associated IR. We evaluated data showing that AT oxidative stress is distinctly regulated in different fat depots and is influenced by ethnicity. As a major source of ROS, especially with increasing obesity, AT function influences whole-body metabolism. VAT accumulation might exert its detrimental effects on metabolic health partly through elevated oxidative stress profile in this depot. However, individuals of African ancestry, despite the relatively lower VAT accumulation, have higher oxidative stress parameters and are at higher risk for IR and T2D than their European counterparts. Their relatively high percentage of SAT (especially gluteal SAT) infer a compromised function of this tissue, which may partly explain their higher rates of IR (in addition to dysfunction in other organs such as muscle, liver or pancreas). Therefore, a higher oxidative state in AT may contribute to the higher susceptibility for metabolic disorders in Africans. However, direct evidence of the relationship between SAT oxidative stress and obesity-associated metabolic risk is lacking. The majority of studies investigating oxidative stress markers in AT were performed among European populations and mainly in abdominal SAT. Furthermore, there are no studies systematically investigating ethnic differences in the associations between AT oxidative stress and metabolic risk factors. Such studies are required to improve our understanding of heterogeneous associations between AT function, obesity-related diseases and the role of oxidative AT stress. In addition, gaining more insights into the ethnic-specific functional difference in the oxidative and inflammatory states of VAT and SAT might be of importance. Finally, further longitudinal and intervention studies involving both African women and men are required to explore AT depot-specific differences in oxidative stress and more importantly, their association with metabolic risk. This would be crucial to gain a better understanding of the potential role and/or influence of AT oxidative stress in the development of metabolic diseases such as IR and T2D in Africans.

## Data Availability

No new data were created or analyzed in this study. Data sharing does not apply to this article.
